# Chloroquine-induced Pruritus

**DOI:** 10.4103/0250-474X.70471

**Published:** 2010

**Authors:** S. E. Aghahowa, H. O. Obianwu, A. O. Isah, I. M. Arhewoh

**Affiliations:** Department of Pharmacology and Toxicology, Faculty of Pharmacy, University of Benin, Benin City, Nigeria; 1 Department of Medicine, School of Medicine, University of Benin, Benin City, Nigeria; 2 Department of Pharmaceutics and Pharmaceutical Technology, Faculty of Pharmacy, University of Benin, Benin City, Nigeria

**Keywords:** Chloroquine-Induced pruritus, treatment

## Abstract

Chloroquine-induced pruritus remains one of the most common side-effects in the use of chloroquine in the prophylaxis and treatment of uncomplicated malaria before the advent of artemisinin-based combination therapies. It has been reported to vary from a tolerable to intolerable intensity among susceptible individuals resulting in disruption of treatment and development of resistance to the drug thus leading to therapeutic failures as reported. This scourge is quite challenging due to the complex physiologic mechanism that has not been fully elucidated. Factors observed to be responsible in the induction of pruritus such as age, race, heredity, density of parasitaemia; impurities in formulations, plasmodial specie, dosage form and metabolites have been discussed in this review. Efforts to ameliorate this burden have necessitated the use of drugs of diverse pharmacological classes such as antihistamines, corticosteroids and multivitamins either alone or as a combination. This review is to look into the use of chloroquine retrospectively, and consider its re-introduction due to its safety. Efficacy can be attained if the pruritic effect is resolved.

Chloroquine-induced pruritus has been described as a ‘biting’ or stinging sensation which occurs after some hours of chloroquine administration irrespective of the route of administration. It is not associated with skin lesions or systemic manifestation and may resolve spontaneously within several days after the drug is discontinued[1]. Pruritus is not only experienced with chloroquine but also with some of the other antimalarials, such as halofantrine and amodiaquine which have been reported to cause pruritus in susceptible individuals although with less intensity[2]. Reports of emergence of resistance of *Plasmodium falciparum* to chloroquine in the tropics presents a serious and urgent problem in the prevention and treatment of malaria with chloroquine. Pruritogenic potential of chloroquine has also been reported to substantially reduce its use, and adversely affected the effective control of malaria and possibly contributed to the emergence of chloroquine resistant strains of *Plasmodium falciparum* through inadequate compliance. Furthermore, it has been reported that 1 out of every 2 persons in the tropics itch with varying degree to the use of chloroquine, which resulted in discontinuation of treatment[1]. Treatment failures associated with the use of chloroquine may not necessarily be as a result of the emergence of resistance but more likely due to non-compliance as a result of pruritus. Several studies on the possible factors responsible for chloroquine-induced pruritus have been documented. This review evaluates some of such factors, the possible mechanism by which the pruritus is induced as well as the efforts so far made to manage it and the overall impact on management of malaria with chloroquine.

## PREVALENCE

Chloroquine–induced pruritus has been reported in 50% of dark skinned Africans, Caucasians and African albinos. Dark skinned persons and Caucasians possess similar numbers of melanocytes throughout the body region but the amount of melanin produced in dark skin is greater and moreover, the synthetic pathway, and packaging of melanin granules (in melanosomes) differ greatly from melanin produced in lighter-skin individuals[1]. Furthermore, *in vitro* skin cell studies showed that chloroquine preferentially binds to melanocytes as opposed to keratinocytes (non melanised *in vitro* only). A clear understanding of the mechanism of chloroquine-induced pruritus will guide help in more effective management of malaria with chloroquine.

## MECHANISM

**Fig. 1:**
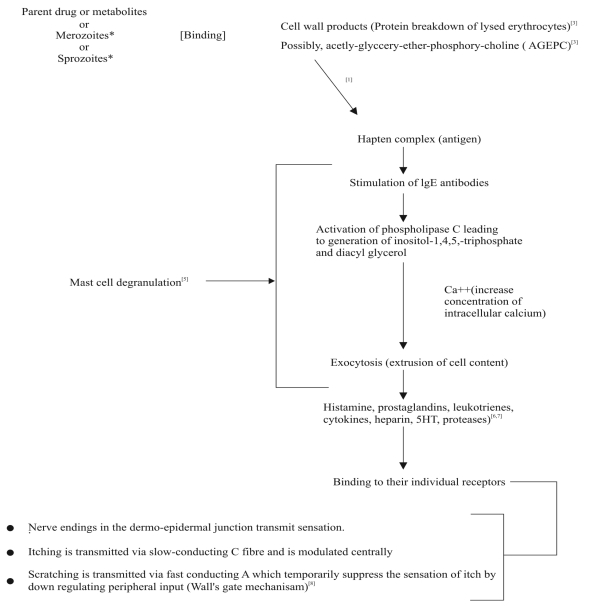
Cascade of events showing possible mechanism of chloroquine-induced pruritus

The possible physiologic processes involved in chloroquine metabolism and mediation of pruritus are basis for suggesting possible mechanisms. It has been suggested that chloroquine or its metabolite (monodesethylchloroquine) act as haptens; binding to break down products of parasitized erythrocytes[3] or possibly interacting with acetyl-glyceryl-ether-phosphoryl-choline (AGEPC), a new class of phospholipids implicated in many aspects of allergy and inflammation[4] (fig. 1). This resulting complex act as an antigen, triggering reagenic antibodies (IgE) that bind to the surface of mast cells (that are in large numbers) and basophils.

These IgE molecules function as receptors and interact with signal transduction system in the membrane of sensitized cell. Upon subsequent exposure, the antigen bridges the IgE molecules and causes activation of phospholipase C, leading to the generation of inositol-1,4,5- triphosphate and diacylglycerol and elevation of intercellular calcium (Ca^2+^). These final events trigger the extrusion of the content of the mast cell granules (degranulation) by exocytosis (fig. 1). The mechanism by which the rise in Ca^2+^ leads to fusion of secretory granules with plasma membrane is not fully elucidated but it likely to involve activation of Ca^2+^/calmodulin-dependent protein kinase and protein kinase C[5]. Several mediators such as histamine, kallikrein, bradykinin, substance P, prostaglandins and various proteolytic enzymes have been implicated in itching[6,7]. These mediators induce or aggravate itch by direct or indirect actions on the sensory nerves and receptors in the skin[8]. A wide variety of mediators released during allergic response may explain the ineffectiveness of a single drug therapy to inhibit multiple mediators.

Another possible mechanism that has been proposed describes a binding of chloroquine or its metabolite to the region of the dermo-epidermal junction which transmit the itching sensation via slow conducting ‘C’ fibres and which are probably modulated centrally. Scratching is transmitted via fast conducting ‘A’ fibers which temporally suppress the sensation peripheral input (wall’s gate mechanism)[8].

It is however possible that these different mechanisms can be at play alone or together in a susceptible individual. One important reason why the mechanism of pruritus is rather complex than what has been illustrated above is the role played by some factors that could be responsible for the induction as well as exacerbation of pruritus, hence influencing the varied response to drug management.

## FACTORS INFLUENCING PRURITUS

### Hereditary factors:

Several studies have suggested that there could be heredo-familial factors influencing chloroquine induced pruritus[2,9,10]. Study has shown that, 133 (40.20%) of 331 patients experienced itching to chloroquine and all those prone to itching to chloroquine did so after oral administration of the drug. Itching was reported to be more common among the families (first degree relations) of itchers but less common among those of non-itchers[9]. Reports of chloroquine–induced itching occurring upon re-administration as observed in 4 patients with a family history, suggest pharmacogenetic factors[1]. Studies have shown that seven patients who itched while on halofantrine gave a personal or family history of itching on chloroquine. The fact that pruritus occurs mainly in blacks and seems to run in families suggests a genetic basis[2]. Identical and fraternal twins have also been reported to have itched, however, this was similar to children of the same parents who do not all itch following chloroquine administration[11]. There was however a difference in the intensity of itch after using chloroquine in malaria therapy. Enzyme deficiency such as glucose-6-phosphate dehydrogenase has been found to be relatively more common among itchers than non-itchers. This implies that G-6-P Dehydrogenase deficiency may perhaps increase susceptibility to chloroquine-induced pruritus. Also studies have shown reduced frequency of the sickle cell trait (HbAS) among itchers compared to non-itchers. This suggests that this trait may be protective against chloroquine-induced pruritus[12]. Difference in the distribution of itchers among the different ABO blood groups has been documented to be insignificant[13].

### Age:

There seems to be a wide variation among various age groups with respect to chloroquine-induced itching. Itching has been reported to start between ages 3 and 5 years. Percentage of individuals prone to itching was observed to increase with age up to 40 years but decreased among senior adults who were above 60 years old[9,14]. In other words, adults between the ages of 20-40 years itched more than children. All these differences may be associated with hepatic metabolism of chloroquine with varied pharmacokinetic disposition with respect to age or different fate of chloroquine metabolism. Some non-susceptible individuals may suddenly become susceptible after the continuous use of chloroquine. This may be associated with repeated exposure to the allergen that may be responsible for itching.

### Racial and skin pigment factors:

Itching has been reported virtually in all races. However, there could be variation in intensity among them. Chloroquine-induced pruritus has been reported in up to 50% of Black-skinned Africans[15–18]. This occurs 6-24 h after administration (oral, parenteral) and lasts for 4-5 days. Itching has been noted to be common in the palms and perineal region. Few individuals have been reported to itched all over their body among Asians[1]. Interestingly, chloroquine and hydroxyl chloroquine have a high affinity for melanin-containing cells, highly abundant in the eyes and skin[15,19,20]. Chloroquine-induced puritus occurs more frequently in black-skinned patients, as opposed to Caucasians or patients with light brown coloured skin such as those of Thailand and ethnic Burmese cohort, suggesting that the degree of skin pigmentation may be an important factor[1]. White and black skin contains similar numbers of melanocytes throughout respective body regions but the amount of melanin granules (in melanosomes) differs greatly from melanin produced in fairer skin[21]. Furthermore, *in vitro* skin cell studies show that chloroquine preferentially binds to melanocytes, as opposed to keratinocytes (non-melanized *in vitro* only)[22]. These factors may contribute to disproportionate accumulation of chloroquine in black skin, in comparison to plasma[23,24].

### Density of parasitaemia:

There have been postulations that pruritus associated with chloroquine administration could be related to the level of parasitaemia. Fifty percent of the inter-subject variation of pruritus intensity is attributable to density of parasitaemia[25]. Some highly prone itchers often report with itching sensation prior to the administration of chloroquine. These findings may be attributed to antibody-antigen reaction at initial exposure of individuals’ immune system to parasites. This notion seems contrary to the absence of chloroquine–induced pruritus in two established itchers, who self–administered chloroquine, despite absence of malaria parasitaemia. Itching prior to the administration of chloroquine could be as a result of the interaction of parasitized erythrocyte with storage sites of itching mediator rather than chloroquine or it metabolites[3]. On the other hand, the itching can result from the interaction of unidentified toxins or unidentified components of lysed erythrocyte with storage vesicles of itching mediators such as histamine and bradykinin[1]. Significant and positive correlations between pruritus intensity and malaria parasite load in itching subjects have also been reported[25].

### Chloroquine metabolites:

Irrespective of the route of administration, chloroquine is rapidly dealkylated via cytochrome P_450_ into pharmacologically active desethyl chloroquine and bis-desethyl chloroquine[26]. Desethyl chloroquine and bis-desethyl chloroquine concentrations reach 50 and 10% of chloroquine concentrations, respectively. Both chloroquine and desethyl chloroquine concentrations decline slowly, with elimination half lives of 20-60 days. Both parent drug and metabolite can be detected in urine months after a single dose[26]. Their peak concentration effect at about eleven hours after administration of chloroquine may be associated with pruritus. Studies have shown that monodesethylchloroquine could be responsible for the pruritus. It is however, possible that pruritus might be due to the interaction of mono-desethyl chloroquine with acetyl glyceryl ether phosphoryl chloride (AGEPC), a new class of potent phospholipids implicated in many aspects of allergy and inflammation that was earlier reported[4]. The mono-desethyl chloroquine which is lipophilic and has affinity for phospholipid like the parent chloroquine has been clearly implicated as responsible for the chloroquine-induced pruritus. The onset of pruritus commencing at about eleven hours after oral administration may be linked with the time the metabolites achieved their peak concentration to elicit itching.

### Mediators:

Several mediators exogenous and endogenous have been identified to elicit itching irrespective of the disease these are bradykinin, prostaglandins, and various neurotrophins (such as neurotrophin-3 and -4) and proteases[6,7]. The use of several drugs of diverse classes to alleviate the itching has yielded varied responses in affected individuals. The variations may be associated with multiple mediators. The use of antihistamines in suppressing itching associated with histamine may have been unsuccessful because other mediators that may potentate or influence the itching whose receptors are not targeted by antihistamines. The wide variety of mediators released during allergic response explains the ineffectiveness of drug therapy focused on a single mediator[5]. Emphasis has been placed on the regulation of mediator released from mast cells and basophiles, and these cells do contain receptors linked to signaling systems that can enhance or block the IgE-induced release of mediators[5]. The contribution or influence of other mediators to itching may be responsible for the failure or ineffectiveness of antihistamines that are solely targeted for histaminergic receptors[17]. Antihistamine trials with corticosteroid may have yielded successful results due to the inhibition of multiple mediators associated with itching.

### Dosage form of chloroquine:

There is a wide variation in the report from individuals taking different chloroquine formulations. Chloroquine base is commonly formulated as sulphate, phosphate and diphosphate. Individuals often report with greater intensity of itching whenever they use the sulphate preparation. Some individuals often report itching to the tablets without itching to the injectable formulation. This suggests that the intensity of chloroquine itching may be reduced by altering the route and form of administration as earlier suggested[27,28]. However, the parenteral preparations do not contain the excipients in the oral dosage form yet intramuscular/intravenous administration of chloroquine does elicit pruritus in susceptible patients.

### Impurities and excipients in commercial preparation:

Various excipients are used in the formulation of chloroquine. They are usually of natural, synthetic and semi-synthetic origin. During the sourcing of the excipients from their origin, impurities are unavoidably included. This may serve as a potential source of contamination, thereby eliciting itching. Also multiple dose injections of chloroquine are preserved with benzyl alcohol BP 1.5% w/v. (BP 2000). These preservatives could serve as substance in chloroquine preparation triggering up itching reactions. Leaching of compounds from containers during storage and compounding are potential sources of contamination. During these processes, impurities may be added unavoidably to chloroquine irrespective of the dosage form. It is possible that these impurities can elicit itching (Personal communication).

### Specie of plasmodium:

Due to wide distribution of plasmodial species, pruritus has been reported in virtually all species of plasmodia. Pruritus has been reported in 1189 individuals resident in Thailand, infected with *P. vivax*[1]. Also, pruritus has been reported in individual infected with *P. falciparum*[25]. Report of pruritus resulting from chloroquine when used in infection related to plasmodia species may be associated with the inherent unidentified substances that are present in the cytoplasm of the parasite.

## APPROACHES IN THE MANAGEMENT OF PRURITUS

The re-evaluation of the effectiveness of antipruritus agent has been difficult due to complex physiologic mechanisms involved in chloroquine induced pruritus. Early studies suggested sedatives and tranquillizers, but these agents have not proved successful because they only sedate individuals making them to be less worried about the itching sensation. Although they still classify the event as being less troublesome, even though they continue to scratch as much as before.

### Antihistamines:

The antihistamines commonly used till-date includes promethazine and chlorpheniramine were reported a partial success rate among the antihistamines on age group between 20 and 24 years who presented with chloroquine-induced pruritus in the out-patient department of the University of Benin Teaching Hospital, Benin City[27]. Promethazine appeared to be the most effective. The differences however, were not statistically significant compared with chlorpheniramine, mepyramine using Chi-square analysis. Furthermore, there was no obvious difference in the mode of administration of chlorpheniramine to 20 patients who presented with chloroquine-induced pruritus. The success rate was only 49% (8 out of 20 patients benefited) with concomitant therapy[28]. There was also report of ineffectiveness of antihistamines using a questionnaire among 550 acutely fibril patients residing or working in the University of Benin, Benin City[10]. A trial was conducted on 23 patients who were infected with *P. vivax* residing in the Thai-Mayanmar border, a malaria endemic area. It was reported that chlorpheniramine or hydroxyzine hydrochloride provided a symptomatic relief[1]. One possible reason why the antihistamines failed is because their protective effect wears away while the chloroquine and its metabolite remains in the blood longer and hence have a tendency to stimulate pruritus. Hence it will be needful to investigate the antipruritic effect taking into cognisance the half-life and therapeutic blood level of the antihistamines which may necessitate a more frequent dosage regimen. Other theories suggested is the substitution of cyproheptadine for ordinary (H_1_) histamine antagonists because of its equally specific antagonism of serotonin and its usefulness in prostaglandin-potentiated itch induced by those amines. Analgesics containing opium alkaloid such as codeine should not be given along with chloroquine in anticipation of itching reaction has been suggested[23]. Alternatively, ammonium chloride mixture 1 gm thrice daily, to acidify the urine and promote a more rapid excretion of chloroquine can alleviate acute itching reaction following chloroquine therapy.

### Corticosteroids:

The use of prednisolone has been advocated compared to promethazine alone in the treatment of pruritus. Prednisolone caused a statistically significant reduction in pruritus compared with promethazine, niacin and the combination of prednisolone and niacin. The finding also showed that histaminergic mechanisms play a small role in chloroquine induced pruritus. They further reported that dosage of prednisolone of 10 mg single dose administered orally yielded a significant result on concurrent prescription with chloroquine in 28 historical itchers. Malaria parasite clearance and clinical amelioration were unaffected by any of the treatment[17].

### Opioid antagonists:

Due to the complex mechanism of pruritus irrespective of the pathogenesis some opioid antagonists have been identified to have antipruritic effect examples of such are µ-opioid antagonists (naloxone and naltrexone) that have been observed in experimentally evoked histamine-induced itch as well as pruritus in different dermatoses[29,30]. Interestingly, µ-opioid receptor antagonists significantly diminish itch, in animal experiments, κ-opioid antagonists enhanced itch[31]. The benefit of opioid receptors in chloroquine-induced itch needs to be elucidated.

### Anxiolytics and sedatives:

The effect of diazepam has been previously assessed[32]. Other studies used clemastine and corticosteroid[33]. There was no significant effect in the use of clemastine and ketotifen when compared to the placebo, while prednisolone significantly reduced the itching. Studies have also reported the use of serotonin type 3 receptor antagonist[34]. Their results suggested that serotonin, acting via 5-HT_3_ receptors, was involved in the generation and/or sensation of pruritus. Therefore, 5-HT_3_ receptor antagonists may be a novel therapeutic principle for the treatment of pruritus.

### Dapsone:

Dapsone or sulphapyridine (500 mg tablets, 6 hourly, orally daily for 5 days) was reported to have palliative effect against chloroquine-induced pruritus in adult patients of age between 16 and 32 years. It was found that dapsone significantly reduced pruritus, but ketotifen and prednisolone did not significantly exceed the placebo responsiveness to vitamin B-complex tablets[18].

## CONCLUSIONS

Only recently the artemisinin-based combination therapies are emerging as first line drugs in the management of uncomplicated malaria. However, it can be predicted that chloroquine will still remain the first line drug in the foreseeable future in malaria treatment primarily because of its cost, safety profile and convenient regimen. As earlier discussed however, pruritus rather than resistance account for the greater proportion of therapeutic failures associated with the use of chloroquine in the management of malaria. While investigations on the mechanisms and management of chloroquine induced pruritus continues, and the possible genetic involvement yet to be fully elucidated, current management of pruritus remains largely individualised, while bearing in mind that effective approach to managing pruritus is essential in the effective treatment of malaria with chloroquine.
